# Microfluidic cell squeeze-based vaccine comes into clinical investigation

**DOI:** 10.1038/s41541-023-00641-x

**Published:** 2023-05-04

**Authors:** Shuhang Wang, Yuqi Yang, Yan Zha, Ning Li

**Affiliations:** 1 Clinical Cancer Center, Cancer Hospital Chinese Academy of Medical Sciences, Beijing, China; 2 NHC Key Laboratory of Pulmonary Immunological Diseases, Guizhou Provincial People’s Hospital, Guiyang, China

**Keywords:** Drug delivery, Cell vaccines, Drug development

## Engineered cell-based therapy face challenges

Cancer immunotherapy has been widely applied to fight various cancers as one of the most successful anticancer therapeutic approaches in recent years. However, the effect of immunotherapy in clinical applications is still unsatisfactory due to the limited response rate and immune-related adverse events. Cell-based drug delivery technology has rapidly emerged as a promising approach to improve the efficacy of cancer immunotherapy potentially, due to its low immunogenicity, prolonged circulation time and superior targeting toward specific tissues^1^. Conventional intracellular delivery approaches, mainly through viral vectors, endocytotic delivery, or mechanoporation methods, face challenges that limit the translational potential of cell engineering therapies. Limitations include imperfect influx mechanisms, incompatibility of cargo size with classical gene editing tools, potential integration of viral sequences into the human genome, reduced primary cell functionality, and off-target perturbations, which may impact therapeutic efficacy and safety^1,2^.

**Fig. 1:**
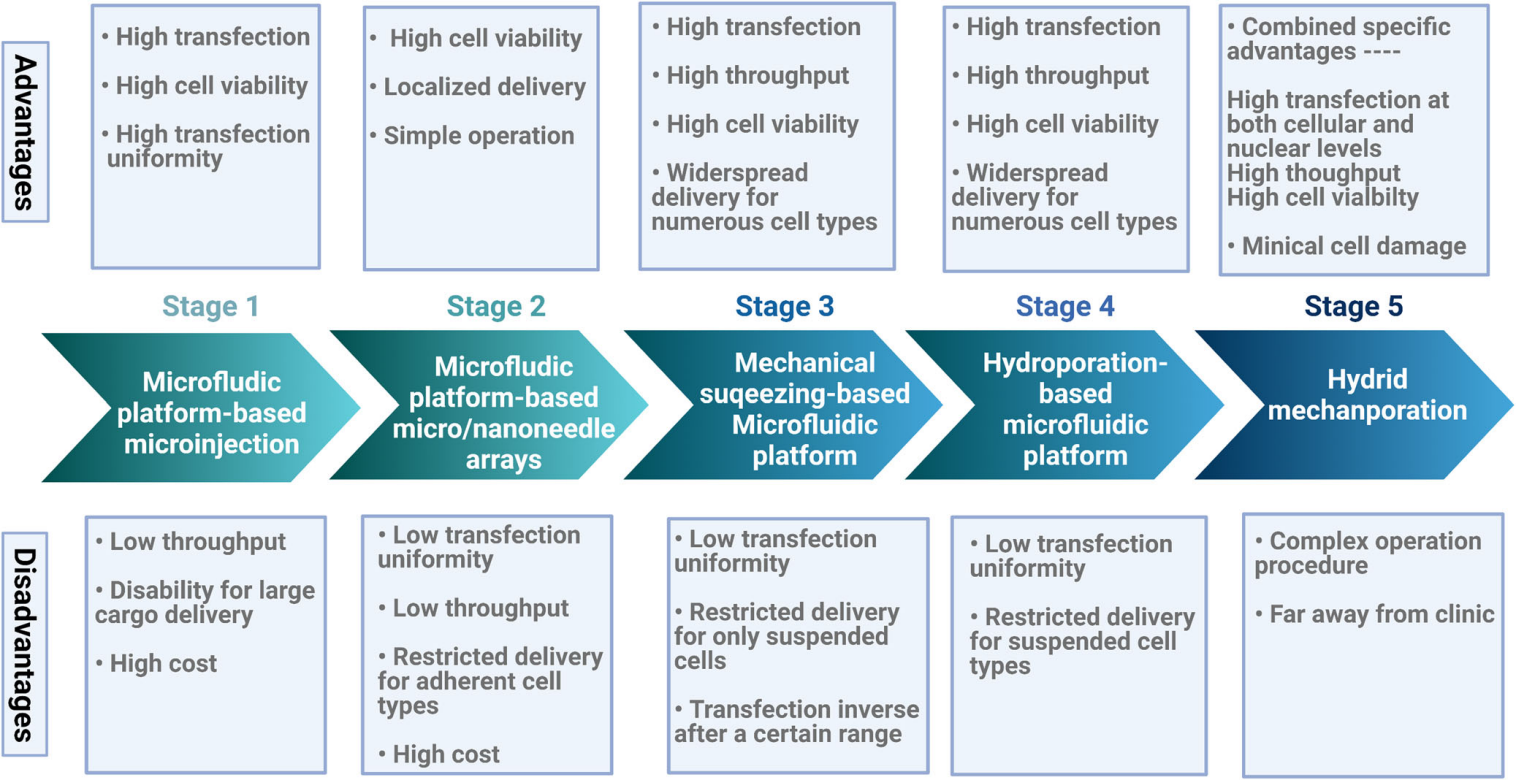
Technologic development of microfluidic mechanoporation for cellular delivery.

A next-generation highly efficient intracellular delivery platform is essential to overcome these limitations and facilitate translation to a broader range of clinical applications. Recent microfluidic platform-based mechanoporation strategies, such as microinjection, micro/nanoneedle arrays, mechanical cell squeezing and hydroporation, have developed rapidly due to high delivery transfection efficiency, throughput and cell viability into numerous cell types (Fig. 1)^2^. The strategies can deliver biomaterials directly into the cytosol of a wide array of cell types mainly base on microfluidics, which refer to a system can deal with a small amount of fluid (10^−9^–10^−18^ L) through tens to hundreds of micrometers of channels^3^. As one of above technologies, mechanical squeezing-based microfluidic platform has entered the clinic phase due to its unique advantages.

## Cell Squeeze technology demonstrates promising advances

**Fig. 2:**
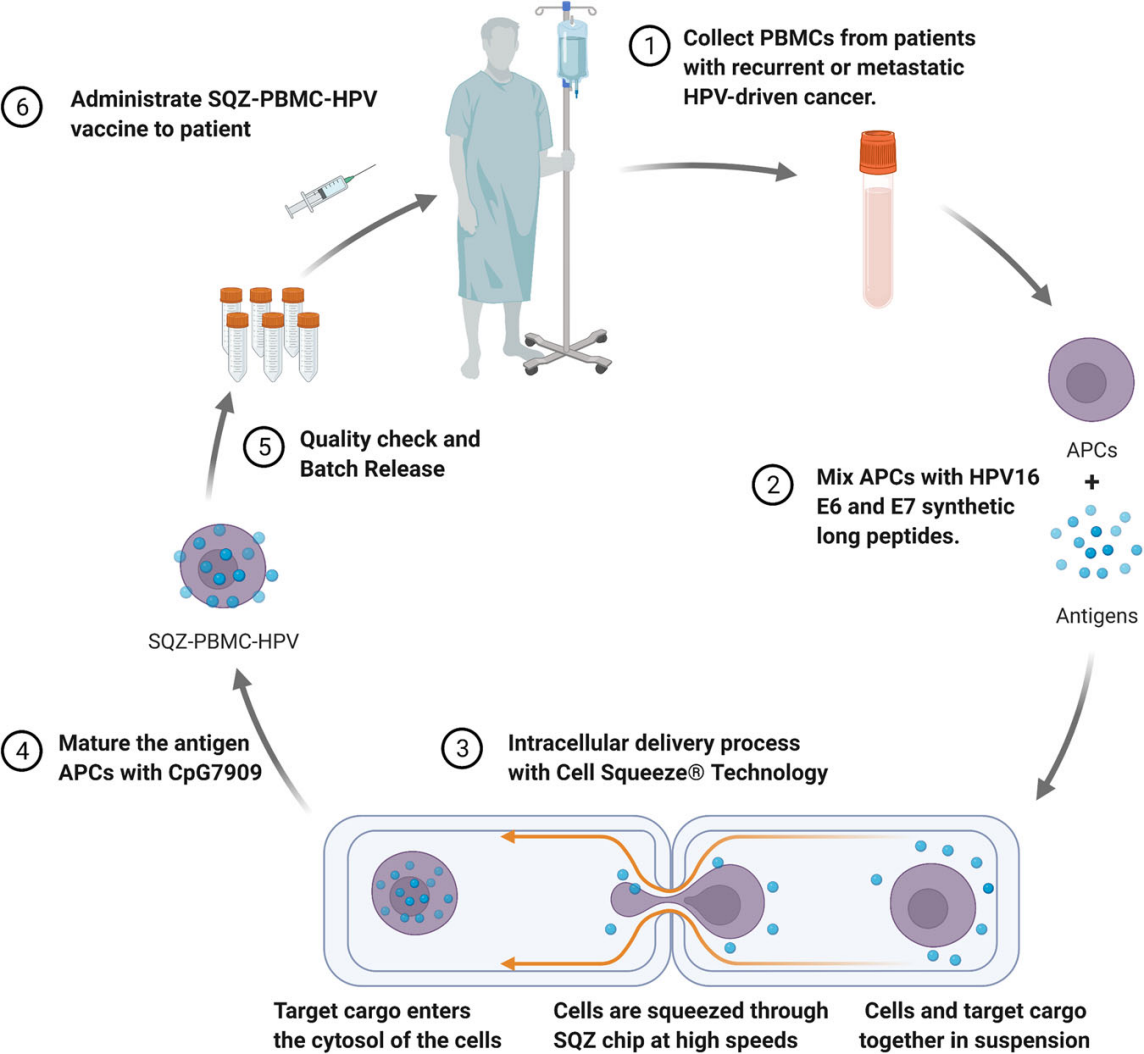
SQZ-PBMC-HPV vaccine production process with Cell Squeeze® Technology.

The microfluidic Cell Squeeze technology has the ability to deliver a variety of biological materials into a variety of cell types. Cell Squeeze platform mainly consists of three major components: a microfluidic chip with multiple channels parallelly, each containing at least one constriction point; a reservoir system to interface with the chip for the collection of cell suspension; and a pressure regulation system to pressurize the reservoirs and facilitate fluid flow through the chip^3,4^. First, cells are resuspended at 10–50 × 10^6^ cells/ml as the delivery buffer. Second, the platform mixes the desired cells with the target delivery material in suspension and loads them into the reservoir system. Third, the mixture is squeezed through relative microfluidic constriction points depending on the cell type being squeezed. Finally, once transient mechanical deformation of cells occurs, temporary pores are generated in the cell membrane to allow for delivery of various biological cargoes directly into the cytosol of cells (Fig. 2).

Several advances in the field of cell-based vaccines have been made as demonstrated by in vitro and in vivo studies. First, the Cell Squeeze technology has demonstrated delivery of cargo to all major cell subsets of human peripheral blood mononuclear cells (PBMCs) with different ranges of cell sizes in a single squeeze process, including T cells, B cells, monocytes and NK cells. Second, direct delivery to the cytosol using this technology enables efficient antigen presentation via major histocompatibility complex I (MHC-I) and therefore potent CD8^+^ T cell activation. Antigen presentation and CD8^+^ T cell activation following the Cell Squeeze process has been shown in animal models to be more than one thousand times as efficient as cross presentation after endocytotic uptake of antigens^5^. In addition, squeezed antigen-loaded antigen presenting cells (APCs) have demonstrated strong antitumor effects in vivo, both prophylactically and therapeutically. Third, the Cell Squeeze technology minimally alters normal cellular function which may benefit its overall safety profile versus conventional intracellular delivery approaches when entering the clinic. Fourth, the process is scalable for clinical trials. Processing of up to 10 billion cells per minute allows for overall cell therapy production to be under 24 h, patients do not require preconditioning, and lengthy hospital stays can be avoided.

## Cell squeeze-based vaccines enter to clinic

Taken together, these advances provide the basis for a potent, rapid turnaround model and highlight the significant clinical potential of the Cell Squeeze platform to advance cell-based vaccines (Table 1). A phase 1/2 clinical trial, SQZ-PBMC-HPV-101 (NCT04084951, from https://clinicaltrials.gov, search on May 1, 2022), a first-in-human, dose-escalation study of a cell-based vaccine, is ongoing for the treatment of HLA*02+ patients with recurrent, locally advanced, or metastatic HPV16 + solid tumors. SQZ-PBMC-HPV is an autologous cell vaccine targeting HPV16 viral oncoprotein E6 and E7 using the Cell Squeeze platform (Fig. 2)^6^. The median time for manufacturing was 17 h and allowed for a vein-to-vein time of about a week. Product characterization confirmed antigen presentation and high viability (median 91%) in all patient batches. 18 patients with various solid tumors (head & neck, anus, cervix) with a median of 3.5 prior lines of systemic cancer therapy were dosed in 4 different dose-escalation cohorts. The initial results from the first three monotherapy cohorts demonstrated that the investigational cell therapy can stimulate immune responses in certain patients. Patients were evaluated for clinical response with paired biopsies of the tumor pre- and on-treatment. On-treatment biopsy of one patient with heavily pretreated, PD-1 inhibitor refractory head and neck cancer in the highest dose cohort found that CD8 tumor infiltration increased and changed the immunophenotype from desert to inflamed, IFN-γ secretion driven by CD8^+^ T cells and HLA-I expression increased, and the frequency of E6 and E7 expressing cells was reduced significantly, all consistent with the proposed mechanism of action of SQZ-PBMC-HPV. In addition, on-treatment biopsy in the same patient showed that PD-L1 expression increased after SQZ-PBMC-HPV treatment, suggesting that SQZ APCs may work synergistically with immune checkpoint inhibitors to provide additional clinical benefit to patients, especially those considered refractory to checkpoint inhibition. Treatment was well tolerated, most related adverse events (AEs) were of low grade at all dose levels, and only one serious AE (SAE), a grade 2 cytokine release syndrome in the lowest dose cohort which was considered related and resolved in <24 h, and no dose-limiting toxicities were observed. The clinical trial has advanced into the combination stage with checkpoint inhibitors targeting the PD-1/PD-L1 and CTLA-4 pathways. Additionally, the highest dose cohort is still enrolling to further characterize safety and efficacy of SQZ-PBMC-HPV as monotherapy.

**Table 1:** Advances studies on microfluidic cell squeeze technology.

Drug name	Cells	Target	NCT	Study stage	Cancer type	Treatment
SQZ-PBMC-HPV-101	Peripheral blood mononuclear cells	HPV16 viral oncoprotein E6 and E7	NCT04084951	I/II	HPV16 + solid tumors	Monotherapy or combined immune checkpoint inhibitors
SQZ-AAC-HPV	Red blood cells	immunogenic epitopes of HPV16	NCT04892043	I	HPV16 + solid tumors	Monotherapy or combined immune checkpoint inhibitors
SQZ-eAPC-HPV	Peripheral blood mononuclear cells	incorporating 5 mRNAs	NCT05357898	I/II	HPV16 + solid tumors	Monotherapy or combined immune checkpoint inhibitors

The same Cell Squeeze platform is also being evaluated in a clinical study in locally advanced or metastatic HPV associated cancers using SQZ activating antigen carriers (AACs), a novel autologous engineered red blood cell (RBC) derived approach to CD8 immunity (NCT04892043, from https://clinicaltrials.gov, search on May 1, 2022). SQZ-AAC-HPV leverages the natural processing of RBCs to target endogenous APCs and potentially generate high quantities of tumor epitope specific CD8+ T cells as well as high quality T cell responses. The first-in-human study is a safety-adjusted monotherapy dose escalation study followed by a combination phase study testing the recommended phase 2 dose in combination with checkpoint inhibitors. This is a global study recruiting in the United States, Spain, and the Netherlands and a clinical trial application has been submitted to the NMPA’s Center for Drug Evaluation.

## Cell squeezing technology need further development

Although mechanical cell squeezing technology is high advantageous, the reduced transfection efficiency for targeting larger biomolecule size or DNA molecules, relative low transfection uniformity, severe cell deformation and a certain possibility of device clogging issue obstructed its further development. Considering the specific advantages of different intracellular delivery systems, hybrid mechanoporation, a combined strategy of mechanical squeezing and other membrane-disruption delivery technique, has attracted many researchers. Combining the high throughput delivery capability of cell squeezing with the efficacy of electroporation for rapid DNA transfection has demonstrated significant improvements when compared to the single method (Fig. 1)^7^. However, an in-depth understanding of combined mechanisms and complex operation procedures still need urgently.

In summary, the Cell Squeeze platform is a promising intracellular delivery technology based on microfluidic mechanoporation, with high transfection efficiency, high cell viability, high throughput, short production timelines, and good safety data. An excellent combination strategy of cell squeeze platform and other delivery techniques can be expected in future research. Currently, Cell Squeeze based vaccine has the potential to provide clinical benefit in a broad range of clinical indications. Based on encouraging initial results in a proof-of-concept clinical trial for HPV associated cancers, successful cell based therapeutic cancer vaccines may eventually become a reality, both as monotherapy or in combination with other cancer treatments, such as checkpoint inhibitors and/or chemotherapy.
